# Supplementary material for: DAZL Knockout Pigs as Recipients for Spermatogonial Stem Cell Transplantation
Source: Cells. 2023 Nov 6;12(21):2582. doi: 10.3390/cells12212582 (PMC10649044; doi:10.3390/cells12212582)
Supplement: Supplementary file 1 [file cells-12-02582-s001.zip › cells-2651753-supplementary.pdf]

DAZL knockout pigs as recipients for spermatogonial stem cell transplantation

<u>(Supplementary Material)</u>

Nathalia L.M. Lara[1], Taylor Goldsmith[2], Paula Rodriguez-Villamil[2], Felipe Ongaratto[2], Staci Solin[2], Dennis Webster[2], Uyanga Ganbaatar[2], Shane Hodgson[2], Stanislas M.A.S. Corbière[2], Alla Bondareva[1], Daniel F. Carlson[2], Ina Dobrinski[1*]

[1] Faculty of Veterinary Medicine, University of Calgary, AB, Canada.

[2] Recombinetics, Inc., St. Paul, MN, USA.

*** Correspondence:**

Ina Dobrinski

idobrins@ucalgary.ca

403-210-6523

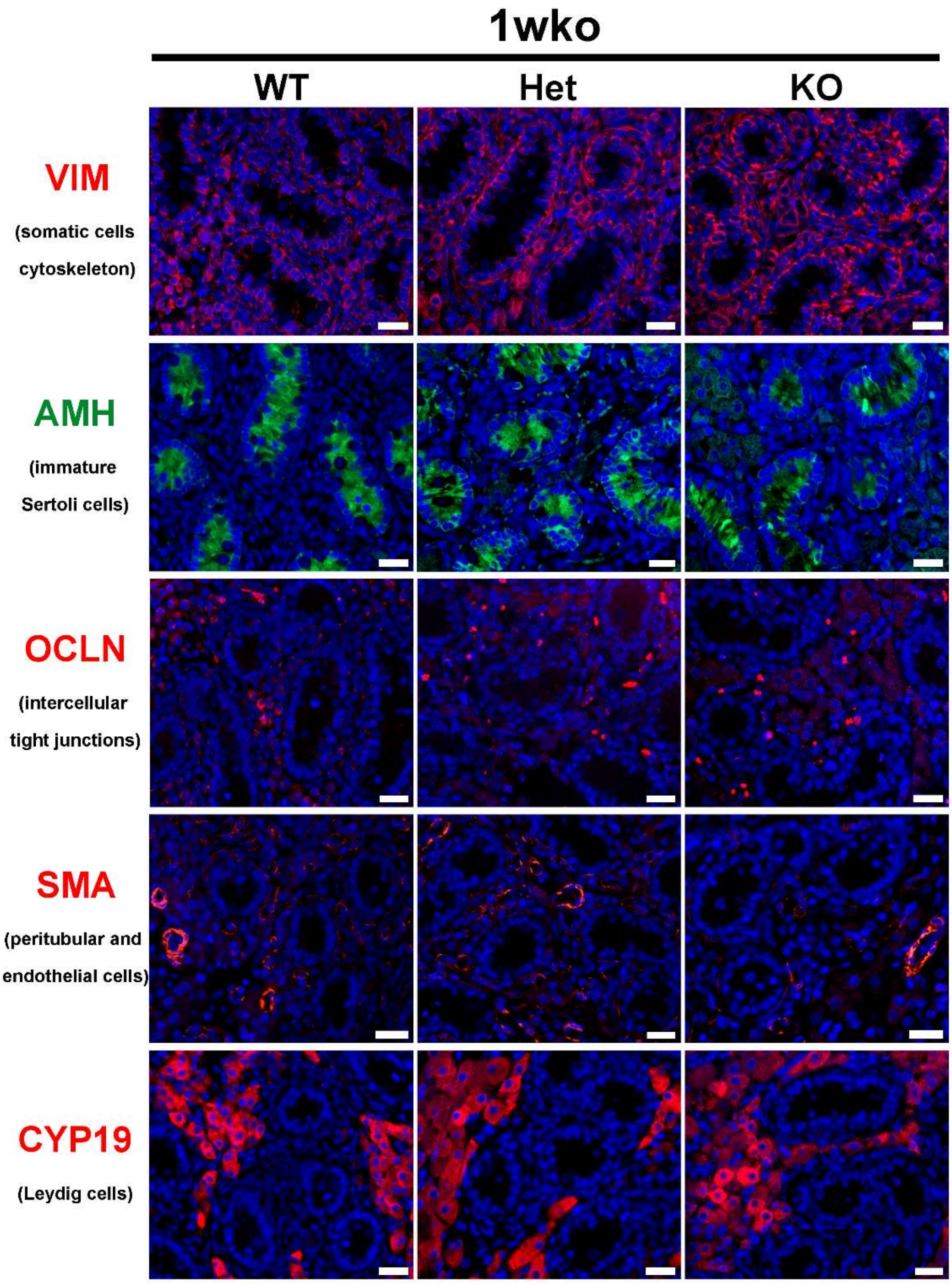

Supplementary Figure S1. The expression of markers of somatic cell function is similar between WT, Het and *DAZL*-KO pig testis at 1 week of age. Vimentin (VIM) is an intermediate filament present in the cytoskeleton of testicular somatic cells, anti-Müllerian Hormone (AMH) is produced by immature Sertoli cells, occludin (OCLN) is an intercellular tight junction protein, alpha-smooth muscle actin (SMA) is expressed by peritubular myoid and endothelial cells, and aromatase (CYP19) is a steroidogenic enzyme present on Leydig cells. Scale bar = 25μm.

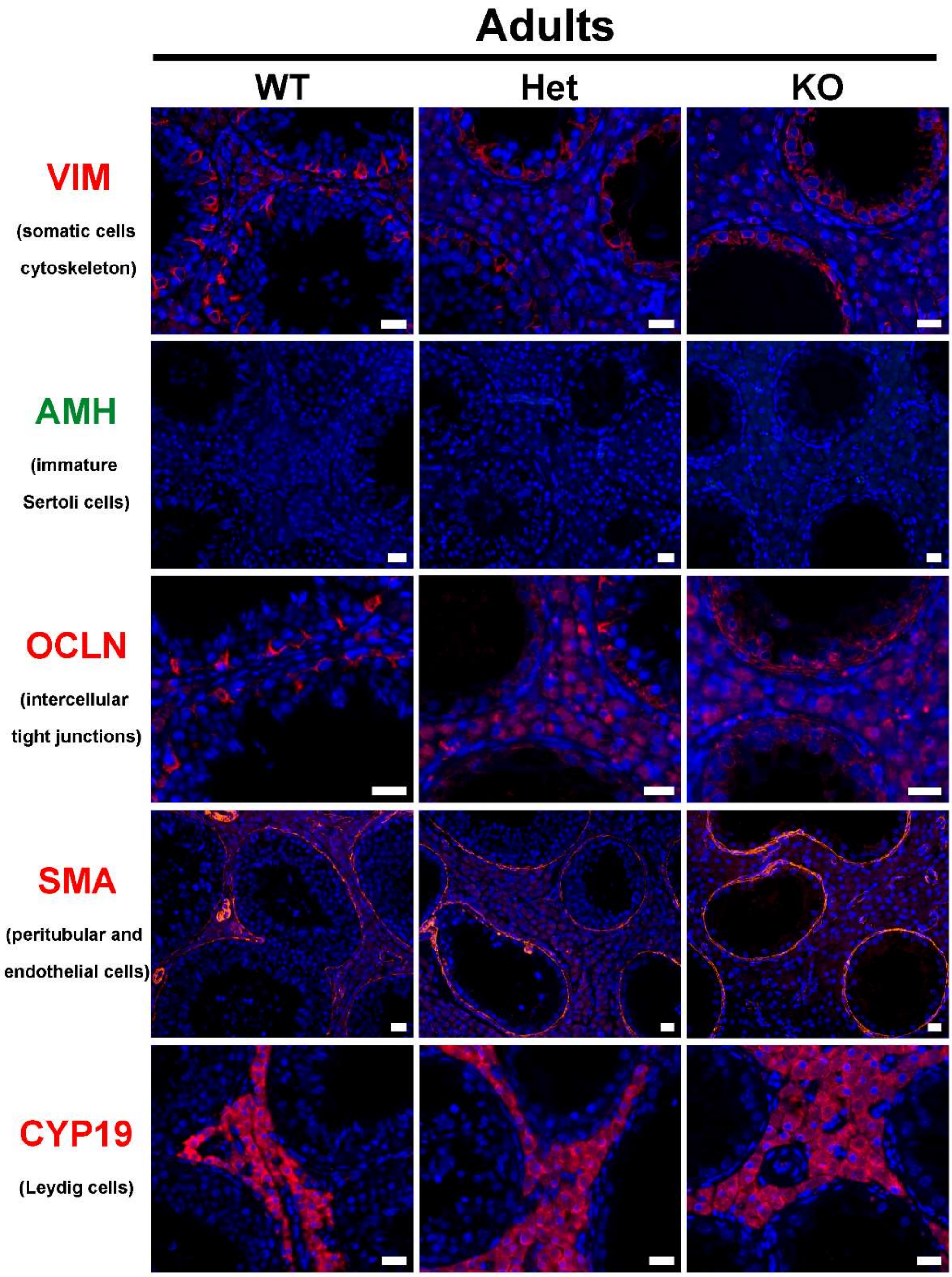

Supplementary Figure S2. The expression of markers of somatic cell function is similar between WT, Het and *DAZL*-KO pig testis at adulthood. Vimentin (VIM) is an intermediate filament present in the cytoskeleton of testicular somatic cells, anti-Müllerian Hormone (AMH) is produced by immature Sertoli cells, occludin (OCLN) is a intercellular tight junction protein, alpha-smooth muscle actin (SMA) is expressed by peritubular myoid and endothelial cells, and aromatase (CYP19) is a steroidogenic enzyme present on Leydig cells. Scale bar = 25µm.

Supplementary Table S1: List of primers used for single nucleotide polymorphisms (SNP) analysis.

SNPs	Alt	Location	Direction	Sequence (5'-3')	Ta
C	T	1071051	Forward<br>Reverse	GCATCTGCCTCTATTTTGTGTT<br>CCATGGCAGGCAGAAGTT	65
C	T	2778674	Forward<br>Reverse	CAGGTGGTGGGAAGAAAAGA<br>ATGCACAGAGGTGCACACA	65
G	T	3144768	Forward<br>Reverse	GCTCTTTGGGAATTCACACTTT<br>TTTGCACATGTCCTGGAGAG	60
C	A	6537618	Forward<br>Reverse	CGGAGTTTTCAGTTGGCTTC<br>TTACGAGGAACAAAGCAGACC	65
A	G	6788853	Forward<br>Reverse	CCTGGAGACCTCAAGCTCCT<br>TACACACAGACGTGCCCCTA	65
G	T	8710551	Forward<br>Reverse	CCCATGCGTTAGTATAGCTGTG<br>CGAAAGCCTCAGGTTCCTTT	65
T	C	15331319	Forward<br>Reverse	GGCAGACAAGCTGTTGTCAC<br>GGGGTGTGTGGGTAACAGAG	65
T	C	24267515	Forward<br>Reverse	CTTCCCATAGCCTGTGCAAT<br>CCGCTTTTGTGAAGTTTTTC	60
T	C	36589903	Forward<br>Reverse	AGCTCGTGATCCTGTTGCTT<br>TATTCTCCTTGCTGGGCTTG	65
T	C	40206250	Forward<br>Reverse	CAGCTACCATGCTGTGAGGA<br>GAGATGACACGAAGGCAAGA	65
T	C	115838003	Forward<br>Reverse	GCCCTAAAGCCAATCCATTT<br>TAAATGCTGGACGCATCAAC	65
A	G	127411174	Forward<br>Reverse	GTTTCCCCTTTTCCTGATCC<br>CTCGGGTGGAGTCTCTGAAG	65
C	T	127491880	Forward<br>Reverse	GGGGGAGTACATGTGGTCTG<br>CATCTACGCATGCCTTTCAA	65
A	G	127725116	Forward<br>Reverse	CCAGAGCCGTTTCATTCCT<br>CAGATACAGCCCGAGCAAAT	65

Ta = Primer annealing temperature.

Supplementary Table S2: List of antibodies used.

Target	Manufacturer	Catalog number	Dilution	Species
UCH-L1	Abcam	ab108986	1:100	Rabbit anti-human
TNP1	Abcam	ab73135	1:100	Rabbit anti-human
PCNA	Dako	M0879	1:100	Mouse anti-rat
VIM	Abcam	ab92547	1:200	Rabbit anti-human
AMH	Santa Cruz	sc-6886	1:400	Goat anti-human
OCLN	Proteintech	66378-1	1:150	Mouse anti-human
SMA	Abcam	ab7817	1:200	Mouse anti-human
CYP19	Bio-Rad	MCA20775	1:400	Mouse anti-human
Alexa Fluor 488	ThermoFisher	A11055	1:500	Donkey anti-goat
Alexa Fluor 594	ThermoFisher	A21203	1:500	Donkey anti-mouse
Alexa Fluor 594	ThermoFisher	A21207	1:500	Donkey anti-rabbit
Alexa Fluor 488	ThermoFisher	A21206	1:500	Donkey anti-rabbit
